# Supplementary material for: Impact of Lachancea thermotolerans on Chemical Composition and Sensory Profiles of Viognier Wines
Source: J Fungi (Basel). 2022 Apr 30;8(5):474. doi: 10.3390/jof8050474 (PMC9146010; doi:10.3390/jof8050474)
Supplement: Supplementary file 1 [file jof-08-00474-s001.zip › jof-1690211-supplementary.pdf]

Table S1. Two-way ANOVA statistics summary for analytical parameters in Viognier wines co-fermented by five *Lachancea thermotolerans* (LT1-LT5) strains, including % of variation described by the LT strain, inoculation regime and their interaction, and their corresponding P-values. Volatile compounds in italics were detected below their sensory threshold in all wines. Compounds in italics and bold were in some wines below and in others above their sensory threshold.

Compounds	LT strain	P-value	Inoculation regime	P-value	Interaction	P-value
Glucose (g/L)	33	0.0183	13	0.0345	14	0.2122
Fructose (g/L)	32	0.0290	5	0.1804	18	0.1835
RS (g/L)	32	0.0280	6	0.1676	17	0.1835
Ethanol (% v/v)	35	0.0032	16	0.0066	20	0.0420
pH	45	<0.0001	29	<0.0001	23	<0.0001
TA (g/L)	39	<0.0001	25	<0.0001	29	<0.0001
Lactic acid (g/L)	39	<0.0001	26	<0.0001	27	<0.0001
Glycerol (g/L)	19	0.0352	35	0.0002	17	0.0622
Acetic acid (g/L)	31	0.0024	2	0.2112	43	0.0011
Malic acid (g/L)	31	0.0009	22	0.0003	27	0.0031
Succinic acid (g/L)	53	<0.0001	24	<0.0001	17	0.0001
Acetaldehyde (mg/L)	39	0.0002	4	0.0977	38	0.2122
Pyruvic acid (mg/L)	34	0.0006	17	0.0010	29	0.1835
Total SO_2 (mg/L)	31	0.0015	17	0.0016	30	0.1835
Ethyl acetate	71	<0.0001	3	0.0595	12	0.0163
Ethyl lactate	*34*	*0.0001*	*19*	*0.0002*	*31*	*0.0006*
Ethyl propanoate	*38*	*<0.0001*	*36*	*<0.0001*	*13*	*0.0123*
Ethyl 2-methylpropanoate	***35***	***0.0001***	***32***	***<0.0001***	***17***	***0.0077***
Ethyl butanoate	57	0.0001	7	0.0479	10	0.1835
Ethyl 2-butenoate	75	<0.0001	7	0.0010	10	0.004
Ethyl hexanoate	51	0.0007	4	0.1540	13	0.1687
Ethyl octanoate	57	<0.0001	5	0.0991	14	0.0716
Ethyl decanoate	**67**	**<0.0001**	**2**	**0.2215**	**9**	**0.1835**
Diethyl succinate	*71*	*<0.0001*	*13*	*0.0010*	*1*	*0.8151*
Σ Ethyl esters	25	0.0005	23	<0.0001	38	0.0001
Isoamyl acetate	64	<0.0001	2	0.1295	19	0.0034
Hexyl acetate	*76*	*<0.0001*	*1*	*0.2975*	*10*	*0.0360*
2-Phenylethyl acetate	*64*	*<0.0001*	*3*	*0.0977*	*17*	*0.0090*
Σ Acetate esters	64	<0.0001	2	0.1246	19	0.0040
1-Propanol	54	0.0025	<1	0.7137	4	0.8139
1-Butanol	*24*	*<0.0001*	*22*	*<0.0001*	*47*	*<0.0001*
Isobutanol	*44*	*0.0009*	*19*	*0.0033*	*9*	*0.2530*
3-Methyl-1-butanol	17	0.1133	4	0.1929	41	0.0087
1-Hexanol	*29*	*0.0097*	*11*	*0.0256*	*30*	*0.0116*
1-Octanol	22	0.0005	45	<0.0001	21	0.0013
2-Phenylethanol	***62***	***<0.0001***	***<1***	***0.7053***	***28***	***0.0001***
Benzyl alcohol	*22*	*0.0154*	*32*	*0.0002*	*19*	*0.0346*
Σ Higher alcohols	21	0.0855	4	0.2112	31	0.0386
Butanoic acid	25	0.0062	44	<0.0001	7	0.2980
Isobutyric acid	*45*	*<0.0001*	*1*	*0.3142*	*37*	*0.0004*
Hexanoic acid	45	<0.0001	28	<0.0001	16	0.0016
Octanoic acid	53	<0.0001	27	<0.0001	14	0.0004
Decanoic acid	73	<0.0001	13	<0.0001	8	0.0059
Σ Acids	54	<0.0001	26	<0.0001	14	0.0005
Limonene	*55*	*<0.0001*	*14*	*0.0004*	*17*	*0.0040*
a-terpineol	*43*	*0.0003*	*14*	*0.0041*	*21*	*0.0134*

Linalool	18	0.3041	<1	0.7726	14	0.4567
Σ Terpenes	30	0.0558	4	0.2975	14	0.3370

Table S2. Qualitative information about the analysed volatile compounds in Viognier experimental wines. Volatile compounds in italics were detected below their sensory threshold in all wines. Compounds in italics and bold were in some wines below and in others above their sensory threshold.

Compound (μg/L)	Compound group [a]	CAS number	Aroma detection threshold (μg/L)	Descriptor	Reference
Ethyl acetate	EE	141-78-6	15000 [b]	VA, nail polish	[4]
Ethyl lactate	*EE*	*687-47-8*	*146000* [b]	*buttery, butterscotch, fruit*	[4]
Ethyl propanoate	*EE*	*105-37-3*	*2100* [c]	*pineapple, fruity*	[2]
Ethyl 2-methylpropanoate	***EE***	***97-62-1***	***15*** [c]	***sweet***	**[5]**
Ethyl butanoate	EE	105-54-4	20 [b]	strawberry	[4]
Ethyl 2-butenoate	EE	10544-63-5	14 [b]	floral	[3]
Ethyl hexanoate	EE	123-66-0	14 [c]	green apple	[5]
Ethyl octanoate	EE	106-32-1	5 [c]	fruity, peach	[5]
Ethyl decanoate	***EE***	***110-38-3***	***200*** [c]	***fruity***	**[5]**
Diethyl succinate	*EE*	*123-25-1*	*1250000* [b]	*wine, fruit*	[4]
Isoamyl acetate	AcE	123-92-2	160 [c]	banana	[5]
Hexyl acetate	*AcE*	*142-92-7*	*670* [b]	*fruity, floral*	[4]
2-Phenylethyl acetate	*AcE*	*103-45-7*	*2400* [c]	*honey, rose*	[5]
1-Propanol	HALC	71-23-8	500 [c]	alcohol, pungent	[4]
1-Butanol	*HALC*	*71-36-3*	*150000* [c]	*fusel*	[1]
Isobutanol	*HALC*	*78-83-1*	*40000* [c]	*solvent, burnt*	[5]
3-Methyl-1-butanol	HALC	123-51-3	30000 [c]	solvent, burnt	[5]
1-Hexanol	*HALC*	*111-27-3*	*8000* [c]	*grassy*	[5]
1-Octanol	*HALC*	*111-87-5*	*0.7* [b]	*chemical, metal, burnt*	[4]
2-Phenylethanol	***HALC***	***60-12-8***	***14000*** [c]	***rose, floral***	**[4]**
Benzyl alcohol	*HALC*	*100-51-6*	*10000* [d]	*fig, tobacco, chocolate*	[1]
Butanoic acid	A	107-92-6	200 [d]	rancid, sweat, cheese	[5]
Isobutyric acid	*A*	*79-31-2*	*2000* [d]	*rancid, butter, cheese*	[5]
Hexanoic acid	A	142-62-1	400 [d]	fatty, rancid, cheese	[5]
Octanoic acid	A	124-07-2	500 [d]	fatty, rancid	[5]
Decanoic acid	A	334-48-5	1000 [d]	fatty, rancid	[5]
Limonene	*T*	*5989-27-5*	*15* [b]	*lemon, orange*	[4]
a-terpineol	*T*	*98-55-5*	*250* [c]	*lilac*	[4]
Linalool	T	78-70-6	15 [c]	floral, lavender	[4]

[a] Abbreviations correspond to the following: EE – ethyl ester, AcE – acetate ester, HALC – higher alcohol, A – acid, T – terpene; Reported thresholds were determined in: [b] hydroalcoholic solution; [c] model wine; [d] multiple matrices, including water, hydroalcoholic solution, beer and wine.

Table S3. Quantitative information about the analysed volatile compounds in Viognier experimental wines sorted by the decreasing mean odour activity value (OAV). Volatile compounds in italics were detected below their sensory threshold in all wines. Compounds in italics and bold were in some wines below and in others above their sensory threshold.

Compound	Minimum (μg/L)	Maximum (μg/L)	Median (μg/L)	Mean (μg/L)	OAV (MIN)	OAV (MAX)

Ethyl octanoate	453	1039	783	745	90.51	207.75
1-Propanol	24268	44086	36807	36988	48.54	88.17
Ethyl hexanoate	492	1141	949	881	35.16	81.53
Octanoic acid	9880	26229	22372	20090	19.76	52.46
Ethyl 2-butenoate	75	354	197	205	5.34	25.27
Ethyl butanoate	221	350	274	282	11.05	17.51
Hexanoic acid	2651	5389	4650	4296	6.63	13.47
Isoamyl acetate	799	2881	1642	1618	4.99	18.01
Butanoic acid	1398	1791	1621	1606	6.99	8.95
1-Octanol	1.4	11.4	5.7	5.6	1.96	16.24
3-Methyl-1-butanol	93603	125227	101657	104330	3.12	4.17
Ethyl acetate	38471	65667	50403	50497	2.56	4.38
a-terpineol	41	63	46	48	2.70	4.18
Decanoic acid	1441	3935	3297	3034	1.44	3.93
Ethyl 2-methylpropanoate	***14***	***19***	***15***	***15***	***0.93***	***1.24***
Ethyl decanoate	***120***	***337***	***197***	***202***	***0.60***	***1.68***
2-Phenylethanol	***11327***	***16232***	***14402***	***14024***	***0.81***	***1.16***
Linalool	*108*	*133*	*124*	*122*	*0.43*	*0.53*
Isobutanol	*15441*	*24093*	*17911*	*18519*	*0.39*	*0.60*
Isobutyric acid	*760*	*1104*	*892*	*920*	*0.38*	*0.55*
Hexyl acetate	*179*	*310*	*244*	*249*	*0.27*	*0.46*
Limonene	*3.9*	*6.1*	*4.5*	*4.7*	*0.26*	*0.41*
1-Hexanol	*1300*	*2560*	*2205*	*2116*	*0.16*	*0.32*
2-Phenylethyl acetate	*272*	*462*	*310*	*329*	*0.11*	*0.19*
Ethyl lactate	*522*	*79647*	*2933*	*11482*	*<0.01*	*0.55*
Ethyl Propanoate	*74*	*100*	*81*	*83*	*0.04*	*0.05*
1-Butnol	*598*	*1029*	*757*	*779*	*<0.01*	*<0.01*
Benzyl alcohol	*43*	*61*	*50*	*50*	*<0.01*	*<0.01*
Diethyl succinate	*15*	*67*	*30*	*32*	*<0.01*	*<0.01*

Table S4. Mean ratings for 33 sensory attributes scored by 48 wine experts. Attributes significantly (ANOVA; Tukey's post-hoc α = 5 %) affected by yeast treatments are in bold with different letters within a row representing significant differences.

Sensory attribute	Yeast treatment												
	SC	LT1xSC	ISVV25…SC	LT2xSC	LT2…SC	CONxSC	CON…SC	LT4xSC	LT4…SC	LT5xSC	LT5…SC	UN	P-value
stone fruit_A	3.63	3.71	3.46	3.29	3.13	3.21	3.40	3.35	3.65	3.52	3.25	3.19	0.9209
banana_A	2.38	2.50	2.33	2.71	2.60	3.04	2.75	2.69	2.60	2.71	2.69	3.10	0.9209
citrus_A	2.63	2.77	3.00	2.83	2.63	3.04	2.88	2.77	2.79	2.58	2.73	2.73	0.9772
apple/pear_A	3.42	3.50	3.50	3.17	3.31	3.19	3.35	3.46	3.08	3.60	3.29	3.02	0.9772
floral_A	2.42	2.27	2.79	2.35	2.69	2.31	2.48	2.50	2.44	2.65	2.69	2.65	0.9772
herbacious_A	1.38	1.10	1.58	1.29	1.17	1.33	1.40	1.10	1.50	1.23	1.02	1.35	0.5787
medicinal_A	1.23	1.13	1.25	1.25	1.04	1.17	1.19	1.02	1.38	1.04	1.21	1.06	0.9772
tropical_A	3.21	2.71	3.06	2.67	3.31	3.17	3.19	3.06	3.06	3.38	2.94	3.35	0.6429
nutty _A	1.31	1.69	1.27	1.46	1.50	1.27	1.29	1.29	1.31	1.29	1.35	1.33	0.9772
honey_A	2.06	2.19	2.23	2.35	2.27	2.21	2.21	2.04	2.29	2.23	2.23	2.29	0.9929
oxidation_A	1.83	1.50	1.77	1.69	1.81	1.77	1.65	1.54	1.63	1.56	1.60	1.77	0.9772
vinegary_A	1.13	1.23	1.29	1.29	1.48	1.13	1.27	1.27	1.08	0.94	1.33	1.17	0.7963
stone fruit_F	3.19	3.15	3.10	3.10	2.77	3.13	3.25	3.27	3.15	3.04	2.96	3.52	0.9772
**banana_F**	**2.31 a**	**2.71 a**	**2 ab**	**2.4 a**	**1.35 b**	**2.52 a**	**2.65 a**	**2.56 a**	**2.56 a**	**2.42 a**	**1.94 ab**	**2.65**	***0.0001***
**citrus_F**	**2.81 bc**	**2.92 bc**	**3 bc**	**2.83 bc**	**4.27 a**	**2.69 bc**	**2.73 bc**	**2.48 c**	**2.96 bc**	**2.69 bc**	**3.48 ab**	**2.48**	**<0.0001**

apple/pear_F	3.04	3.31	2.98	2.92	3.06	3.08	2.85	3.21	3.44	2.98	3.10	3.10	0.9772
floral_F	2.29	2.40	2.52	2.21	1.75	2.23	2.02	2.50	2.31	2.27	2.13	2.40	0.4278
herbacious_F	1.48	1.35	1.65	1.79	1.54	1.50	1.75	1.29	1.52	1.44	1.31	1.33	0.5787
medicinal_F	1.31	1.23	1.04	1.35	1.17	1.38	1.54	1.27	1.29	1.38	1.21	1.08	0.9772
tropical_F	2.67	2.85	2.81	2.67	2.83	2.79	2.81	2.71	3.02	2.69	2.94	3.13	0.9772
nutty _F	1.10	1.27	1.15	1.17	1.02	1.50	1.17	1.21	1.21	1.17	0.96	1.54	0.9662
honey_F	2.04	1.90	2.25	2.02	1.56	2.10	2.10	2.10	2.21	1.77	1.79	2.27	0.4891
oxidation_F	1.73	1.88	1.69	1.79	1.65	1.98	1.88	1.67	1.58	1.33	1.50	1.85	0.7590
vinegary_F	1.40	1.23	1.19	1.33	1.81	1.06	1.17	1.27	1.21	1.02	1.38	1.27	0.1354
**sweetness**	**3.85 a**	**3.63 ab**	**3.46 abc**	**3.67 ab**	**2.94 c**	**3.48 abc**	**3.69 a**	**3.65 ab**	**3.6 ab**	**3.27 abc**	**3.08 bc**	**4.08**	**<0.0001**
**acidity**	**3.69 c**	**3.73 c**	**4.08 bc**	**3.75 c**	**5.15 a**	**3.71 c**	**3.65 c**	**3.52 c**	**3.73 c**	**4 bc**	**4.54 ab**	**3.69**	**<0.0001**
bitterness	3.23	3.38	3.00	3.19	3.00	3.31	3.60	3.19	3.31	3.31	3.08	3.06	0.4278
hotness	3.63	3.48	3.63	3.77	3.40	3.75	3.69	3.54	3.90	3.79	3.54	3.63	0.4891
body	3.79	3.77	3.96	3.85	3.56	3.83	3.81	4.00	3.88	3.71	3.90	3.88	0.5831
**balance**	**3.56 ab**	**3.77 ab**	**4.08 a**	**3.63 ab**	**3.27 b**	**3.63 ab**	**3.4 b**	**3.54 ab**	**3.81 ab**	**3.46 b**	**3.83 ab**	**3.69**	***0.0023***
fruit flavour length	3.94	3.85	4.04	4.02	3.96	3.58	3.69	3.83	4.15	3.85	4.13	4.21	0.4278
non-fruit flavour length	3.67	3.67	3.71	3.79	3.69	3.77	3.77	3.75	3.88	3.90	3.58	3.77	0.9772
**acidity length**	**3.46 c**	**3.96 bc**	**4 bc**	**3.71 bc**	**5.17 a**	**3.56 c**	**3.63 bc**	**3.56 c**	**3.98 bc**	**3.88 bc**	**4.27 b**	**3.60**	**<0.0001**

Table S5. Frequency of responses (%) for acidity profiles of Viognier wines fermented with 12 yeast treatments. The yeast treatments include the *Saccharomyces cerevisiae* monoculture (SC), five *Lachancea thermotolerans* strains (LT1-LT5) in co-inoculations (xSC) or sequential inoculations (…SC) with *S. cerevisiae*, and an un-inoculated treatment (UN).

Yeast treatment	Acidity profile			
	Flat/flabby	Crisp/fresh/bright	Sour/tart	Harsh/acrid
SC	24	14	5	5
LT1xSC	17	16	13	2
LT1…SC	6	26	14	2
LT2xSC	15	21	10	2
LT2...SC	2	13	29	4
LT3xSC	21	16	7	4
LT3...SC	17	12	12	7
LT4xSC	18	16	11	3
LT4...SC	15	21	8	4
LT5xSC	14	19	7	8
LT5...SC	2	25	16	5
UN	19	16	8	5

References

1. Cameleyre, M.; Lytra, G.; Tempere, S.; Barbe, J.-C. Olfactory Impact of Higher Alcohols on Red Wine Fruity Ester Aroma Expression in Model Solution. *J. Agric. Food Chem.* **2015**, *63*, 9777–9788, https://doi.org/10.1021/acs.jafc.5b03489.
2. Lytra, G.; Tempere, S.; Le Floch, A.; De Revel, G.; Barbe, J.-C. Study of Sensory Interactions among Red Wine Fruity Esters in a Model Solution. *J. Agric. Food Chem.* **2013**, *61*, 8504–8513, https://doi.org/10.1021/jf4018405.
3. Slegers, A., Angers, P., Ouellet, E., Truchon, T., & Pedneault, K. Volatile Compounds from Grape Skin, Juice and Wine from Five Interspecific Hybrid Grape Cultivars Grown in Quebec (Canada) for Wine Production. *Molecules*, **2015**, *20*, 10980–11016.
4. Wang, J.; Capone, D.L.; Wilkinson, K.L.; Jeffery, D.W. Chemical and sensory profiles of rosé wines from Australia. *Food Chem.* **2015**, *196*, 682–693, https://doi.org/10.1016/j.foodchem.2015.09.111.
5. Waterhouse, A.L.; Sacks, G.L.; Jeffery, D.W. *Understanding Wine Chemistry*, 1st ed.; Wiley: Hoboen, NJ, USA, 2016, https://doi.org/10.1002/9781118730720.
